# Assessment and Management of Depression and Anxiety in Children and Adolescents with Epilepsy

**DOI:** 10.1155/2019/2571368

**Published:** 2019-05-02

**Authors:** David Plevin, Nicholas Smith

**Affiliations:** ^1^Cramond Clinic, The Queen Elizabeth Hospital, Woodville South, South Australia 5011, Australia; ^2^School of Medicine, The University of Adelaide, Adelaide SA 5005, Australia; ^3^Department of Neurology, Women's and Children's Hospital, North Adelaide SA 5006, Australia

## Abstract

Anxiety and depression in children and adolescents with epilepsy are common comorbidities which place a significant burden on patients and families and complicate the clinical management of epilepsy. This paper presents a narrative review on the aetiology, phenomenology, assessment, and management of depression and anxiety among paediatric patients with epilepsy. The recognition of affective comorbidity in paediatric epilepsy is limited at present, and the contributory role of antiepileptic medication towards such comorbidity must be considered by clinicians.

## 1. Introduction

In developed countries, the incidence of childhood epilepsy ranges from 33.3 to 82 cases per 100,000 persons per year. The prevalence is much higher, lying between 3.2 and 6.3 cases per 1,000 persons [1]. A significant proportion of these children will experience either anxiety or depression. In clinic-based studies, the prevalence of anxiety in children with epilepsy is around 30 to 35%, while the prevalence of depression is around 12.7 to 36.5% [2]. The burden of psychiatric comorbidity in children and adolescents with epilepsy is significant, leading to increased morbidity and impact on patients and their families. It is also clinically significant, as it complicates the management of epilepsy. Clinicians may not recognise the complex relationship between psychiatric symptoms, epilepsy, and medications used to treat it. Any additional medications that are prescribed to manage psychological symptoms, aside from having inherent individual risk profiles, may further contribute to drug burden and drug interactions with existing antiepileptic medication regimens. In addition, the side effects of antiepileptic drugs themselves may contribute to neurobehavioural and psychiatric outcomes in this population.

## 2. Factors Contributing to Anxiety and Depression in Paediatric Epilepsy

### 2.1. The Psychological Sequelae of Chronic Illness

Chronic illness itself can lead to anxiety and depression. This may be symptomatic of the illness itself; a consequence of the course and management of the illness, leading to a sense of lack of control and uncertainty or hopelessness and helplessness from poor prognoses; an increasing fear of death; stigma and ostracism from peers; overprotective parental behaviour; and side effects from treatment [3, 4]. Overall, the rates of anxiety in paediatric chronic illness have been reported as between 7 and 40% [5]. In insulin-dependent diabetes mellitus, for example, around 20% of children have anxiety disorders and over 25% have major depression [6]. One meta-analysis reported higher rates of most forms of psychopathology in children with epilepsy, including anxiety/depression, compared to healthy children. The magnitude of the difference was markedly smaller between children with epilepsy and other children with chronic illnesses. Notably, however, in comparison to other children with chronic illness, the effect sizes were larger for attention, social, and thought problems, suggesting that these issues, rather than anxiety and depression, may be more specific to epilepsy [7]. These findings indicate that the psychiatric consequences of epilepsy may, in part, relate to the chronic disease burden itself; however, these results may also reflect confounding, as children with neurocognitive deficits may be harder to screen for affective symptoms. This is discussed in further detail below.

### 2.2. Organic Contributions

In most studies, no relationship has been found between the type of seizure and the development of anxiety or depressive symptoms [2], with the possible exception of temporal lobe epilepsy. In a cohort of children and adolescents with either cognitive difficulties or who were considered as potential candidates for surgical management of intractable epilepsy (*n* = 132, age range: 6 to 18), anxiety, depression, and withdrawal were assessed using a caregiver questionnaire—the Behavior Assessment System for Children 2: Parent Rating Scale (BASC-2: PRS). Patients with temporal lobe epilepsy had a statistically significant increase in mean depression scores compared to those with frontal lobe epilepsy (*p* < 0.01). There was no difference between temporal and frontal lobe epilepsy groups for measures of anxiety or withdrawal. Within the subset of children with partial epilepsy, there were no differences in affective symptoms based on seizure laterality, and, in the cohort as a whole, there was no difference in affective symptoms between those children with partial epilepsy and those with generalised epilepsy [8]. Another cohort of children with diagnoses of complex partial seizures and childhood absence epilepsy (*n* = 171, age range: 5 to 16) reported that children with complex partial seizures—essentially the same patient population as those with temporal lobe epilepsy—have a higher rate of depression and comorbid depression and anxiety disorders and a lower rate of anxiety disorders (*p* < 0.02), when compared to childhood absence epilepsy, a common generalised epilepsy of childhood. In this study, the Kiddie Schedule for Affective Disorders and Schizophrenia (K-SADS) for School-Age Children, Epidemiologic Version (K-SADS-E) or Present and Lifetime Version (K-SADSPL) was used to determine the presence of affective, anxiety, and disruptive disorders [9]. Similarly, in the adult literature, there are indications that temporal lobe epilepsy is related with psychopathology [10–12]. The possible relationship between complex partial seizures/temporal lobe epilepsy and interictal psychiatric symptoms may implicate a limbic pathology in the development of these symptoms. Indeed, in animal studies, electrical or chemical stimulation of limbic structures, such as the hippocampus and amygdala, have led to emotional changes similar to anxiety [13].

### 2.3. Pharmacotherapy

Certain individual antiepileptic agents may be associated with adverse psychiatric effects in children. Phenobarbital, for example, has been associated with much higher rates of major depressive disorder and suicidal ideation than carbamazepine [14]. Levetiracetam approximately doubles the risk of behavioural problems in children, including aggression, hostility, and nervousness [14]. Similarly, in adult populations, there is a documented risk of psychiatric adverse effects with certain antiepileptic medications, such as vigabatrin, topiramate, and levetiracetam. Vigabatrin, for example, compared to placebo, has been associated with a greater than threefold increased incidence of depression (2.5% versus 0.3%, *p* < 0.05) and a greater than eightfold increased incidence of psychosis (12.1% vs 3.5%, *p* < 0.001). In controlled trials, between 17 and 28% of patients on topiramate developed “abnormal thinking,” and, in comparison studies, episodes of psychosis occurred at a much higher rate in those patients receiving topiramate compared to those receiving gabapentin or lamotrigine [15].

It is prudent to consider the adverse effect profile when selecting antiepileptic drugs. Although neurobehavioural adverse effects have been reported with all antiepileptic drugs, neurobehavioural effects are less common with certain agents, such as lamotrigine or oxcarbazepine [16].

The use of antiepileptic agents in combination is also associated with neurobehavioural adverse effects. A recent systematic review has reported that while the use of antiepileptic polytherapy is widespread, it is not supported by high-quality evidence backing its efficacy. There were no identified randomized controlled trials assessing the efficacy or neurobehavioural safety of polytherapy. Furthermore, several observational studies indicated that polytherapy has statistically significant associations with depression, anxiety, and behavioural disturbances [17].

## 3. Phenomenology of Depression and Anxiety in Paediatric Epilepsy

In the overall population with epilepsy, affective symptoms may be temporally related to seizure occurrence. It has been reported that, in paediatric patients, pre-ictal symptoms of depression include aggression, irritability, and motor hyperactivity [18].

Previous reviews suggest that the typical features of depression in adults, such as low mood and melancholic features, including disturbed sleep and appetite, anhedonia, and psychomotor retardation, are rarely present in children. Instead, irritability and negative cognitions about self, others, and the world are seen [18, 19]. Irritability and aggression may also be presenting features of anxiety in children [18].

There is a paucity of literature exploring the phenomenology of anxiety and depression in paediatric epilepsy. Furthermore, in the paediatric epilepsy population, one practical difficulty that arises is the question of diagnosing psychiatric symptoms in those children and adolescents with intellectual disability. For example, while there are a limited number of assessment tools for anxiety in children with intellectual disability, it has been noted that some of these tools lack sufficient evaluation as a measure of anxiety, and, for other tools, it is not known whether there can be extrapolation from assessment of anxiety symptoms to the diagnosis of anxiety disorders [20]. In addition, a cohort study of adolescents (*n* = 50) with mild and moderate intellectual disability found no correlation between three different assessment scales of depression, two of which were informant-reported and the other one was self-reported [21]. These difficulties in diagnosis are compounded by the paucity of research in assessing psychological symptoms in children with comorbid epilepsy and intellectual disability.

## 4. Management

The cornerstone of management is vigilant recognition and active monitoring for psychiatric morbidity in children and adolescents with epilepsy. This is particularly critical in patients at a possible higher risk for such morbidity, such as those with temporal lobe epilepsy [8, 9] and those receiving polytherapy [17]. As previously noted, the diagnosis of psychiatric morbidity in those patients with intellectual disability presents a particular difficulty. Assessing affective symptoms in children with intellectual disability is challenging and often overlooked: an outcome which is reflected in the paucity of research assessing the frequency and origin of these symptoms amongst children with epilepsy. Indeed, there is a clear imperative to improve recognition of neurobehavioural symptoms in children and adolescents with comorbid epilepsy and intellectual disability so that clinicians may provide appropriate support and management.

Clinical assessment for psychological symptoms should be part of regular clinical follow-ups. Proactive management options, which may include providing additional supports or formal specialist psychiatric review, may be beneficial for those patients who are at particular risk of developing psychopathology and their families and caregivers. Screening tools for psychiatric comorbidity, where available, may be profitably incorporated into routine clinical assessment. For example, for adolescents with epilepsy, a recently available screening tool based on self-report—the Neurological Disorders Depression Inventory-Epilepsy for Youth (NDDI-E-Y)—has been shown to have reliability, construct validity, and a sensitivity of 79% and a specificity of 92% for the assessment of depressive symptoms [22].

Appropriate management of antiepileptic medication is another crucial component in the psychiatric management of the paediatric epilepsy population. Such management has a number of components: first, clinicians should aim for the judicious selection of antiepileptic drugs with a lower likelihood of psychiatric adverse effects, acknowledging the importance of balancing such considerations against the primary objective of seizure control. Second, prescribers must regularly review and optimise the antiepileptic regimen prescribed to their patients and recognise that stability of effect may not confer stability of adverse drug consequences. This is particularly the case for adverse neurobehavioral symptoms, where contributing factors are often multiple. Consideration must also be given toward the accumulative impact of polypharmacy in this context and should be avoided where possible and minimised when required [17].

Finally, cognitive-behavioural therapy has shown promise in the management of mood symptoms in epilepsy. In one study of adolescents with newly diagnosed epilepsy and what was described as “subthreshold depression”, the group receiving short cognitive-behavioural interventions had statistically significant lowering of scores in the Beck Depression Inventory and Centre for Epidemiological Study on Depression scales (*p* < 0.05 for each). However, there were no statistically significant differences in the number of episodes of depression between the cognitive-behavioural intervention and the treatment as usual groups [23].

## 5. Conclusions

Recognition of psychiatric comorbidity in children and adolescents with epilepsy remains limited and must be improved upon, if we are to achieve a meaningful reduction in the burden of these symptoms on patients and their families. While rarely singularly causative, the impact of anticonvulsant medication should be considered and appropriately addressed by clinicians in this context.

## References

[1] Camfield P., Camfield C. (2015). Incidence, prevalence and aetiology of seizures and epilepsy in children. *Epileptic Disorders*.

[2] Reilly C., Agnew R., Neville B. G. R. (2011). Depression and anxiety in childhood epilepsy: a review. *Seizure*.

[3] Pinquart M., Shen Y. (2011). Anxiety in children and adolescents with chronic physical illnesses: a meta-analysis. *Acta Paediatrica*.

[4] Pinquart M., Shen Y. (2011). Depressive symptoms in children and adolescents with chronic physical illness: an updated meta-analysis. *Journal of Pediatric Psychology*.

[5] Pao M., Bosk A. (2011). Anxiety in medically ill children/adolescents. *Depression and Anxiety*.

[6] Dantzer C., Swendsen J., Maurice-Tison S., Salamon R. (2003). Anxiety and depression in juvenile diabetes: a critical review. *Clinical Psychology Review*.

[7] Rodenburg R., Stams G. J., Meijer A. M., Aldenkamp A. P., Deković M. (2005). Psychopathology in children with epilepsy: a meta-analysis. *Journal of Pediatric Psychology*.

[8] Schraegle W. A., Titus J. B. (2017). The relationship of seizure focus with depression, anxiety, and health-related quality of life in children and adolescents with epilepsy. *Epilepsy and Behavior*.

[9] Caplan R., Siddarth P., Gurbani S., Hanson R., Sankar R., Shields W. D. (2005). Depression and anxiety disorders in pediatric epilepsy. *Epilepsia*.

[10] Gaitatzis A., Trimble M. R., Sander J. W. (2004). The psychiatric comorbidity of epilepsy. *Acta Neurologica Scandinavica*.

[11] Manchanda R., Schaefer B., McLachlan R. S. (1996). Psychiatric disorders in candidates for surgery for epilepsy. *Journal of Neurology Neurosurgery and Psychiatry*.

[12] Perini G. I., Tosin C., Carraro C. (1996). Interictal mood and personality disorders in temporal lobe epilepsy and juvenile myoclonic epilepsy. *Journal of Neurology Neurosurgery and Psychiatry*.

[13] Depaulis A., Helfer V., Deransart C., Marescaux C. (1997). Anxiogenic-like consequences in animal models of complex partial seizures. *Neuroscience and Biobehavioral Reviews*.

[14] Halma E., De Louw A. J. A., Klinkenberg S., Aldenkamp A. P., Ijff D. M., Majoie M. (2014). Behavioral side-effects of levetiracetam in children with epilepsy: a systematic review. *Seizure*.

[15] Schmitz B. (2006). Effects of antiepileptic drugs on mood and behavior. *Epilepsia*.

[16] Afzal K. I., Anam S., Hunter S. J. (2017). The effects of antiepileptic drugs on pediatric cognition, mood, and behavior. *Journal of Pediatric Epilepsy*.

[17] Plevin D., Jureidini J., Howell S., Smith N. (2018). Paediatric antiepileptic polytherapy: systematic review of efficacy and neurobehavioural effects and a tertiary centre experience. *Acta Paediatrica*.

[18] Barry J. J., Ettinger A. B., Friel P. (2008). Consensus statement: the evaluation and treatment of people with epilepsy and affective disorders. *Epilepsy and Behavior*.

[19] Devinsky O. (2003). Psychiatric comorbidity in patients with epilepsy: implications for diagnosis and treatment. *Epilepsy and Behavior*.

[20] Reardon T. C., Gray K. M., Melvin G. A. (2015). Anxiety disorders in children and adolescents with intellectual disability: prevalence and assessment. *Research in Developmental Disabilities*.

[21] Masi G., Brovedani P., Mucci M., Favilla L. (2002). Assessment of anxiety and depression in adolescents with mental retardation. *Child Psychiatry and Human Development*.

[22] Wagner J. L., Kellermann T., Mueller M. (2016). Development and validation of the NDDI-E-Y: a screening tool for depressive symptoms in pediatric epilepsy. *Epilepsia*.

[23] Martinović Z., Simonović P., Djokić R. (2006). Preventing depression in adolescents with epilepsy. *Epilepsy and Behavior*.

